# The Treatment of Tabetic Ataxia by Methodical Exercises

**Published:** 1906-09

**Authors:** Maurice Faure

**Affiliations:** Ancien interne de la clinique Charcot (Salpêtrière), Médecin de l'Institut de la Malou (Hérault)


### THE TREATMENT OF TABETIC ATAXIA BY METHODICAL EXERCISES.

BY

MAURICE FAURE, M.D.

*Ancien interne de la clinique Charcot (Salpêtrière), Médecin de l'Institut de la Malou (Hérault).*

It was in 1890 that Frenkel, of Heiden, announced at the congress of German physicians of Bremen the first cases of improvement obtained in the treatment of ataxy of the upper limbs by means of exercises, graduated and suitably regulated. During the following years (1890 to 1896) the same author obtained analogous results in ataxia of the lower limbs. In Germany at the clinic of Professor Leyden of Berlin, in France at La Salpêtrière in the clinic of Professor Raymond, this practice was carried out with success on the method indicated by Frenkel, and has since been tried a little in most places. These methods are very simple and easily understood: they are based on the facts that writing, walking, grasping, swimming, &c., are exercises acquired by learning, and afterwards by an effort of attention and memory, the subject, who has lost co-ordination of the upper or lower limbs, can re-learn writing, walking, grasping, swimming, &c., if his muscular and nervous systems are not too seriously damaged. Of course, in most cases the patient will not accomplish these acts with the same precision as the normal man, still, in the end he will accomplish them, and this is a great advance.

I gave at the Salpêtrière, in 1896, a first series of statistics in collaboration with Frenkel and Hirschberg. These statistics enabled me to state at once that every ataxic patient affected with inco-ordination of the lower or upper limbs, sufficiently patient to carry out a long and elaborate treatment, sufficiently intelligent to understand the rationale of this treatment, and also capable of the indispensable mental effort to learn and to repeat properly the movements which are its foundation, should have attained a result so considerable that in two to four months his social status is notably changed.

At the same time I sought, in collaboration with Frenkel, to explain the genesis of the motor troubles of ataxics. We came to the conclusion that their attitude and their abnormal movements were due to three factors:—

1. The disturbances of the muscular sense, through which the patient loses the sense of attitude, and is hindered from controlling at all times the position and the movements of his limbs or of his body.

2. Muscular atony. In the normal state the muscular tone, by maintaining as it does a certain degree of contractility and elasticity of itself, keeps the limbs in certain attitudes which they cannot go beyond. In the tabetic the muscle which has been stretched does not return of its own accord to its former length, it is in a state of permanent relaxation, and offers no longer any opposition to attitudes which would be impossible in the normal condition. Thus, with a normal subject lying on the back, a lower limb raised above the plane of the bed (the other limb remaining in contact with the bed) makes, with the axis of the trunk, an obtuse angle. With certain neuropathic women, with infants, with professional acrobats, this angle can be exceeded. With tabetics the limb can, without any effort, be placed at a right angle, or at an angle even more and more acute until the leg is able to touch the face. This is a typical example of what muscular atony and loss of muscular sense may produce. From these two causes the muscular contractions become abrupt and so irregular in force, that they miss their aim and produce the jerking movement of ataxia.

3. Inco-ordination. This is a complex phenomena resulting in great part, as Frenkel and I have thought, from the loss of muscular sense and of the muscular atony, but in part also of compensatory movements which the ataxic himself makes in trying to carry out the normal movements (Pierret), and further in part also to the supervention of attention, of intelligence, and of memory (zone corticale, Jendrassik, Raymond, Grasset) in the carrying out of movements which in the normal state are automatically performed. The co-ordination and the inco-ordination are not, therefore, entirely cerebral, neither are they solely sensory and muscular.

These considerations give us an idea of the meaning of the facts brought forward by Frenkel, and teach us to look for their explanation in a re-education of the muscular sense. This re-education the ataxic unconsciously begins himself when he seeks to make up for his incapacity by ill-directed movements. Our part is to guide and help him along this path by teaching him exercises which help him to carry out again his lost movements, wholly or in part, in the shortest time and with the least labour.

It is with this view that I have endeavoured since that time to devise a code of movements which, while corresponding with all the anomalies which are to be met with in the ataxic, permit him to regain, as quickly and surely as possible, his equilibrium, walking, and all the motor functions of the limbs, the head, and the body.

In making these investigations I have first of all noted several facts which, in addition to those already given by

Frenkel, increase their range. Here are the two most important of these facts:—

1. It is not only the incoordinate movements of the arms and legs which can be corrected in the ataxic by appropriate exercises, but also (and this appears difficult to understand) the disturbances of respiration, of micturition, and of defæcation, the laryngeal crises, the defects of sensation, the sense of equilibrium and of position. In fact, respiration is accomplished by the action of muscles of the neck, chest, and abdomen, which while capable of acting without the control of the will can nevertheless become subordinate to it, and can modify according to its dictates the rhythm and the amplitude of their movements. A special education will then allow the ataxic who has lost the habit of breathing normally, and who is for this reason exposed to anæmia, troubles of nutrition, bronchial infections (and even to fatal disturbances of the broncho-pulmonary circulation), to regain a more normal type of respiration and also to modify his life and improve the prognosis of his disease. The troubles of micturition in tabes are due in great measure to inco-ordination of the diaphragm, the abdominal walls, and of the perineal floor, to arhythmia of the sphincters, all things which special education of movements of these muscles can in a great measure correct. Again, the child learns by education of the sensori-motor tracts to control micturition, to make it voluntary when it was not so, to accomplish it when the need is not felt, and reciprocally not accomplish it when the need is felt, all things which are not innate and natural. Similarly is it with defæcation. The laryngeal crises are due for the most part to the inco-ordination of the muscles of respiration and to the defects of laryngeal sensation, and it is possible to re-educate those muscles and their sensibility, for the re-education of sensibility is possible just as much as the re-education of motility, and for the same reasons. The new-born infant does not clearly perceive muscular sensations, he has not the exact sensation of attitudes, the notion of degree of displacement in space, all things which education teaches him. With the tabetics sensory education is naturally rendered difficult by the presence of lesions of the sensory tracts. It is necessary to take into account the relative integrity of the pure motor tracts, because this makes it easier to understand how re-education of movement is effected. Further, the re-education of muscular sense and of the sense of position cannot attain the regularity and certainty of motor education, but still it is possible to a certain degree. As to the sense of equilibrium, it is simply the resultant of the education in movement of the limbs and body, of the muscular sense, and of the sense of position.

These conclusions are of very great importance in the prognosis of tabes. In fact, the tabetic does not die, so to speak, of his disease; he dies of the complications produced by it. Sometimes he becomes a morphinomaniac from excess of pains, or abuses some other analgesic which ruins his constitution; sometimes insufficiency of micturition brings vesical stasis and urinary infection; obstinate constipation brings digestive troubles and stercoral auto-intoxication; laryngeal crises may themselves be fatal; in fact, we have seen many times tabetics die of broncho-pulmonary infection (tubercular or otherwise) due to imperfect respiration, and which thoracic exercises, well carried out, would certainly have prevented. To sum up, if one succeeds (and that is possible with exercises and special care) in keeping respiration, defæcation and micturition in a normal condition, the health of the tabetic may be prolonged almost indefinitely, so to speak, whilst formerly cachexia, infection, death were the ordinary termination of these thoracic and abdominal complications, almost inevitably fatal in the history of tabes.

The second conclusion which we have been able to reach in the course of these ten years of research is this:—

To teach an ataxic to walk again numerous proceedings are at our disposal. There exist, in fact, many types of gait, but they are not all equally simple, equally logical, equally easy. It is certain that in the beginning of re-education it was thought advisable (and Frenkel himself does not escape this criticism, at least up to 1900) to re-establish the equilibrium and walking in the tabetic by a process at the same time too simple and too severe, and too evidently inspired either by a sort of conception of an ideal walk or by the imitation of the military march. Now these two models are both defective, they lead to exercises which are exasperating, too complicated and useless. I have succeeded in combining in one system the re-education in walking with that of equilibrium and maintenance of movements of the trunk, and the walk that I teach is effected in such a manner that whilst insuring the maximum of stability with the minimum of effort it serves at the same time and with slight modifications for walking up- or down-hill, on the level, or on irregular ground, all points which the ideal walk and the military march fail to accomplish. From which results a very great economy in the efforts of the patient and the teacher, which is appreciable if one thinks that precisely the principal obstacle to re-education of a tabetic is the difficulty of the exercises and the length of the treatment.

So then, taking as our point of departure the work done in collaboration with Frenkel ten years ago, and in making divers important additions and modifications, I have fixed on a method for the compensatory treatment of the ataxia of tabetics, taking into consideration not only the motor disturbances of the arms and the legs, but also the inco-ordination of the trunk, the defects of sensibility, the insufficiency of inspiration, of micturition and defæcation, the difficulties in equilibration, and the sense of position, and completely modifying the health and duration of the life of the tabetic, the prognosis and the evolution of tabes. We shall study in a future paper to what extent each of these disturbances can be ameliorated (for they are capable of relief to a very variable extent), the time necessary to obtain the greatest improvement, which points may be considered as settled as the result of my six years of assiduous practice and the divers statistics which I have published, what still remains to be investigated, and finally what is the complete method and what are the principal technical processes which are employed.

